# Collective invasion in ductal and lobular breast cancer associates with distant metastasis

**DOI:** 10.1007/s10585-017-9858-6

**Published:** 2017-09-11

**Authors:** Antoine A. Khalil, Olga Ilina, Pavlo G. Gritsenko, Peter Bult, Paul N. Span, Peter Friedl

**Affiliations:** 1 Department of Dermatology and Graduate School of Life Sciences, University of Würzburg, Würzburg, Germany; 2 Department of Cell Biology, Radboud University Medical Center, 6500 HB Nijmegen, The Netherlands; 3 Department of Pathology, Radboud University Medical Center, Nijmegen, The Netherlands; 4 Department of Radiation Oncology, Radboud University Medical Center, Nijmegen, The Netherlands; 5 David H. Koch Center for Applied Genitourinary Cancers, The University of Texas MD Anderson Cancer Center, Houston, TX 77070 USA; 6 Cancer Genomics Centre, 3584 CG Utrecht, The Netherlands

**Keywords:** Breast cancer, Collective invasion, Adipose tissue, Epithelial-to-mesenchymal transition, Cell–cell junctions, E-cadherin, CD44, Distant metastasis

## Abstract

Breast cancer undergoes collective tissue invasion and, in experimental models, can collectively metastasize. The prevalence of collective invasion and its contribution to distant metastasis in clinical disease, however, remains poorly defined. We here scored the adipose tissue invasion of primary invasive ductal carcinoma (IDC), expressing E-cadherin, and E-cadherin negative invasive lobular carcinoma (ILC) and identified predominantly collective invasion patterns (86/86 samples) in both carcinoma types. Whereas collective invasion in IDC lesions retained adherens junctions, multicellular clusters and “Indian files” in ILC, despite the absence of adherens junctions (AJ) proteins E-cadherin and β-catenin, retained CD44 at cell–cell contacts. By histomorphological scoring and semi-automated image analysis, we show that the extent of collective invasion into the adipose tissue correlated with decreased distant metastasis-free survival (5-year follow-up; hazard ratio: 2.32 and 2.29, respectively). Thus, collective invasion represents the predominant invasion mode in breast cancer, develops distinct junctional subtypes in IDC and ILC, and associates with distant metastasis, suggesting a critical role in systemic dissemination.

**Electronic supplementary material:**

The online version of this article (doi:10.1007/s10585-017-9858-6) contains supplementary material, which is available to authorized users.

## Introduction

Metastatic progression of breast cancer is initiated by cancer cells traversing the basement membrane of the epithelium of origin, followed by migration through the peritumoral stroma until they enter blood vessels, circulate and seed at distant sites [1–3]. In epithelial cancers, invasion and metastatic progression can proceed by distinct cellular mechanisms, including single-cell and collective invasion [4–6]. In mouse models for breast cancer in vivo both individual and clustered circulating tumor cells (CTCs) can be isolated from peripheral blood and both are able to colonize distant organs [6, 7]. Likewise, individual and clustered CTCs are present in the peripheral blood of stage IV breast cancer patients [7]. Whereas the relevance and mechanisms of collective invasion and metastasis are being uncovered in mouse models, the prevalence of collective invasion in clinical breast cancer, and whether the extent of collective invasion correlates with metastatic progression, remain unexplored.

Whether cells move individually or collectively is defined by the molecular organization and stability of cell–cell junctions [8]. Individual cell invasion results from the downregulation of cell–cell junctions in response to activation signals, e.g. as part of epithelial-to-mesenchymal transition (EMT), whereas collective invasion and metastasis critically depend upon intact junctions between cancer cells, particularly adherens junctions (AJ) and desmosomal adhesions [8, 9]. In mouse models for invasive ductal carcinoma (IDC) and 3D analysis of selected human IDC samples, multicellular invasion is associated with E-cadherin expression along cell–cell junctions, whereby partial E-cadherin downregulation and upregulation of EMT markers were noted in small subregions of collective invasion [10, 11]. This indicates molecular variability of collectively invading cancers.

Despite its emerging relevance for tissue penetration and metastasis, a topologic and molecular classification of collective invasion in clinical breast cancer, its molecular subtypes and prevalence is missing. Using quantitaive 2D and 3D image cytometry in a retrospective cohort of 111 clinical breast cancer samples, we here derive the prevalence of collective invasion in IDC and ILC lesions and its association with metastasis. We show that collective invasion, irrespective of E-cadherin expression, is the default invasion program the extent of which strongly correlates with metastatic outcome.

## Methods

### Antibodies

The following antibodies were used: anti-human E-cadherin (MA5-14458, Thermo Scientific); anti-human E-cadherin (SHE 78-7, Thermo Scientific); anti-human vimentin (chicken polyclonal, Abcam); anti-mouse β-catenin (14/β-catenin, BD Biosciences); anti-pan keratin (C11, CST); anti-human CD44 (rabbit polyclonal, Sigma-Aldrich); secondary Alexa-fluor-488/647-conjugated goat anti-mouse and anti-rabbit and anti-chicken IgG (Invitrogen).

### Primary breast cancer samples

Paraffin-embedded, formalin-fixed breast cancer samples (N = 111) were selected from a retrospective cohort of breast cancer patients collected between January 1991 and December 1996 based on whether patients had experienced distant metastasis within 5 years after primary surgery (N = 48) or not (N = 63) [12]. The two groups of patients (without and with distant metastasis) were stratified for equal frequency of axillary lymph node metastasis (Supplementary Table 1; Table 1). This matched procedure minimizes the otherwise positive association of lymph node metastasis and distant metastasis found in cohorts of randomly selected patients and thus eliminates data bias incurred by this potentially confounding prognostic parameter. All patients received local surgery, mastectomy (N = 57) or lumpectomy (N = 54) and the majority subsequently received radiotherapy (N = 87) and/or adjuvant systemic therapy (N = 24) according to the standard of care at that time (Table 1). Tumor samples were encrypted and analyzed in an anonymized manner, as approved by the institutional review board and according to national law [13].

**Table 1:** Patient subgroups and tumor characteristics

Groups	LNM-free and DM-free	LNM-free and DM	LNM and DM-free	LNM and DM	N
Menopausal status					
Pre	5	10	10	10	35
Post	27	12	21	16	76
Surgical resection					
Lumpectomy	24	12	10	8	54
Mastectomy	8	10	21	18	57
Post-resection therapy					
None	32	22	14	19	87
Endocrine	0	0	8	4	12
Chemo no anthracyclines	0	0	2	0	2
Chemo with anthracyclines	0	0	1	0	1
Chemo and endocrine	0	0	6	3	9
Tumor size					
<2.3 cm	26	17	11	3	57
>2.3 cm	6	4	18	23	51
ND	0	1	2	0	3
Bloom–Richardson grade					
1 and 2	12	12	9	7	40
3	10	7	15	16	48
ND	10	3	7	3	23
N	32	22	31	26	111

*LNM* lymph node metastasis, *DM* distant metastasis, *ND* not determined

### Immunohistochemistry and immunofluorescence of thin tissue sections

Formalin-fixed paraffin-embedded breast tissue sections (5 μm thickness) were deparaffinized, followed by antigen retrieval using Tris–EDTA buffer (95–100 °C) and incubation with 3% hydrogen peroxide at room temperature (RT). Tissues were incubated with anti-E-cadherin antibody (1 h) followed by biotinylated secondary antibody (1 h), streptavidin-horseradish peroxidase (30 min) and DAB substrate solution (5 min). For nuclear staining, tissues were incubated with haematoxylin (1 min). Sections were embedded in xylene-based mounting medium and automatically scanned with a 0.24 μm/pixel resolution (Pannoramic 250 Flash II scanner). For immunofluorescence staining, non-specific epitopes were masked with 5% normal goat serum (NGS) and 0.05% Tween-20 in 1× Tris-buffered saline (TBS), followed by incubation with primary antibody (β-catenin, 1:100; vimentin, 1:400; pan-cytokeratin, 1:200; CD44, 1:300) for 18 h at 4 °C, washed with TBS and incubated with secondary Alexa Fluor-conjugated antibodies (1:400) and DAPI (1 μg/ml) (1 h, RT). After washing, sections were embedded in Fluoromount-G^®^ (Southern-Biotech) and scanned with confocal microscopy (Olympus FV1000) using long working distance 20× NA 0.50 and 40× NA 0.80 objectives with 2.5 μm z-step size, or 20× objective with a resolution of 0.5 μm/pixel using the automated Vectra Intelligent Slide Analysis System (Version 2.0.8, PerkinElmer Inc.).

### 3D reconstruction of thick tissue sections

Tissue sections (thickness: 200 μm) were obtained by sectioning of formalin-fixed breast cancer samples. Antigen retrieval and non-specific epitope masking were performed as for immunofluorescence analysis of thin slices. Tissue slices were incubated for 24 h at room temperature (RT) with anti-E-cadherin (1:100), or anti-CD44 (1:100) antibodies. After each incubation step, thick samples were extensively washed (3–5×, 24 h, RT) followed by incubation (24 h, RT) with secondary antibodies and DAPI (1 µg/ml). 3D confocal reconstructions (Olympus FV1000) were obtained using long working distance objectives 20× NA 0.50 and 40× NA 0.80 with a z-step size of 2.5 µm and digital post-processing (Imaris V.6.1.5 software, Bitplane).

### Quantification of vimentin and cytokeratin levels

For image segmentation and quantification, regions of interest from individual images or image stacks containing normal breast ducts or multicellular cancer groups within the marginal adipose tissue were identified in IDC and ILC tissue sections. Image analysis from both spectrally unmixed epifluorescent images (Vectra 2.0.8, PerkinElmer Inc.) or maximum intensity projections from 3D confocal stacks were manually segmented, background corrected, and the mean gray values of vimentin and cytokeratin were obtained using ImageJ (ImageJ; 1.40v; National Institute of Health) from the following regions: luminal epithelium of normal cytokeratin-positive ducts in the tumor-free margin; multicellular epithelial cytokeratin-positive groups in the marginal adipose tissue; and cytokeratin-negative vimentin-positive stromal cells which were further identified by elongated morphology and spindle-shaped nuclei.

### Quantification of collective invasion by pathological scoring

The peritumor fibrous tissue (collagen-/fibroblast-rich tissue) and the marginal adipose tissue were scored from hematoxylin and E-cadherin stained sections by a board-certified breast cancer pathologist (P.B.) for the presence of multicellular tumor nests, clusters and strands relative to individualized tumor cells. As threshold for positivity for collective invasion, the fraction of cancer cells with multicellular organization located in the fibrous or adipose peritumor tissue was at least 95% in both fibrous and marginal adipose tissue for IDC; 75% in fibrous tissue and 90% in marginal adipose tissue for ILC samples.

Pathological scoring was validated by quantitative analysis of cytokeratin positive events in the peritumor region in a randomly selected subset of IDC (N = 12) and ILC (N = 10) samples (Supplementary Table 2). Unmixed epifluorescent images (Vectra 2.0.8, PerkinElmer Inc.) or maximum intensity projections from 3D confocal stacks of peritumor regions (696 µm × 520 µm and 635 µm × 635 µm, respectively) from 5 µm thick samples were analyzed for the relative frequency of collective versus individual cell invasion patterns using ImageJ (ImageJ; 1.40v; National Institute of Health). For that, the number of pan-cytokeratin positive cells within the peritumor region, either located within a multicellular group or as individual cells without obvious cell neighbor in the same section, was quantified for each sample and expressed as the percentage of all pan-cytokeratin positive cells in the invasion zone.

To determine whether collective invasion correlates with distant metastasis, the extent of collective invasion within the marginal adipose tissue was scored and correlated with the metastasis-free survival. Of the tissue blocks that contained adipose tissue (N = 102), the majority showed adipose tissue invasion (N = 86). The extent of such collective invasion was determined by a board-certified breast pathologist (P.B.) in a blinded fashion from samples stained by anti-E-cadherin antibody and hematoxylin using bright-field microscopy. Cancer cells were identified by their typical histopathological features, including large and irregular-shaped nuclei, invasive growth pattern and, for IDC lesions, E-cadherin positivity. A histopathological collective invasion (CI) score was obtained using the following formula:

$${\text{CI score in adipose tissue}}=\frac{{\% {\text{ of tumor cell area occupying adipose tissue}}}}{{\% {\text{ of adipose tissue in the whole section}}}}$$

First, the percentage of adipose tissue content in the whole section was determined by estimating the proportion of the adipocyte-rich regions relative to the total area of the tissue section, including tumor, fibrous and adipose tissue. The percentage of adipose tissue per sample was comparable in both patient subsets with or without distant metastasis (Supplementary Fig. 3a). This precludes selection bias originating from uneven representation of the stromal compartment.

Then, the area occupied by the tumor cell invasion zone located within the adipose tissue was estimated as percentage of the total tumor area present in each slide. As an example, 10% invading tumor cells located within the adipose tissue indicates that the other 90% of the tumor cells in this slice were located within the fibrous tissue or, non-invading, in the tumor core. The CI score was then obtained as the ratio of the percentage of cancer cells occupying the fat tissue relative to the total adipose tissue area per sample.

### Quantification of collective invasion by semi-automated image analysis

To validate histopathological scoring results, quantitative cytometry was performed on all E-cadherin positive samples (N = 75). The area fraction of adipose tissue relative to the total tissue content was calculated after manual selection, followed by automated thresholding of the E-cadherin channel and manual exclusion of normal ducts, to identify tumor cells expressing E-cadherin in the adipose tissue. The area of collective invasion patterns or tumor cell groups was measured relative to adipose tissue area and relative to the absolute area occupied by E-cadherin positive tumor cell groups within the adipose tissue.

### Statistical analysis

Distant metastasis-free survival was correlated with the CI score, area of the collective invasion zone and tumor size using the medians as cutoff values. Comparison of the Kaplan–Meier curves between patient cohorts with high or low collective invasion and the calculation of the hazard ratio were performed using the Log-Rank test. One-way ANOVA (Kruskal–Wallis test) with multiple comparison test (Dunn’s test) was used to compare vimentin levels in luminal epithelium, cancer groups and stromal cells. CI-scores or areas occupied by collective invasion between patient subsets were compared using the Mann–Whitney test. We have not applied multivariate analyses, based on considerations of the relatively low sample number and the pre-stratification for either metastatic or non-metastatic outcome. All statistical analyses were performed using the GraphPad Prism software (version 5). P values below 0.05 were considered as statistically significant.

## Results

**Fig. 1:**
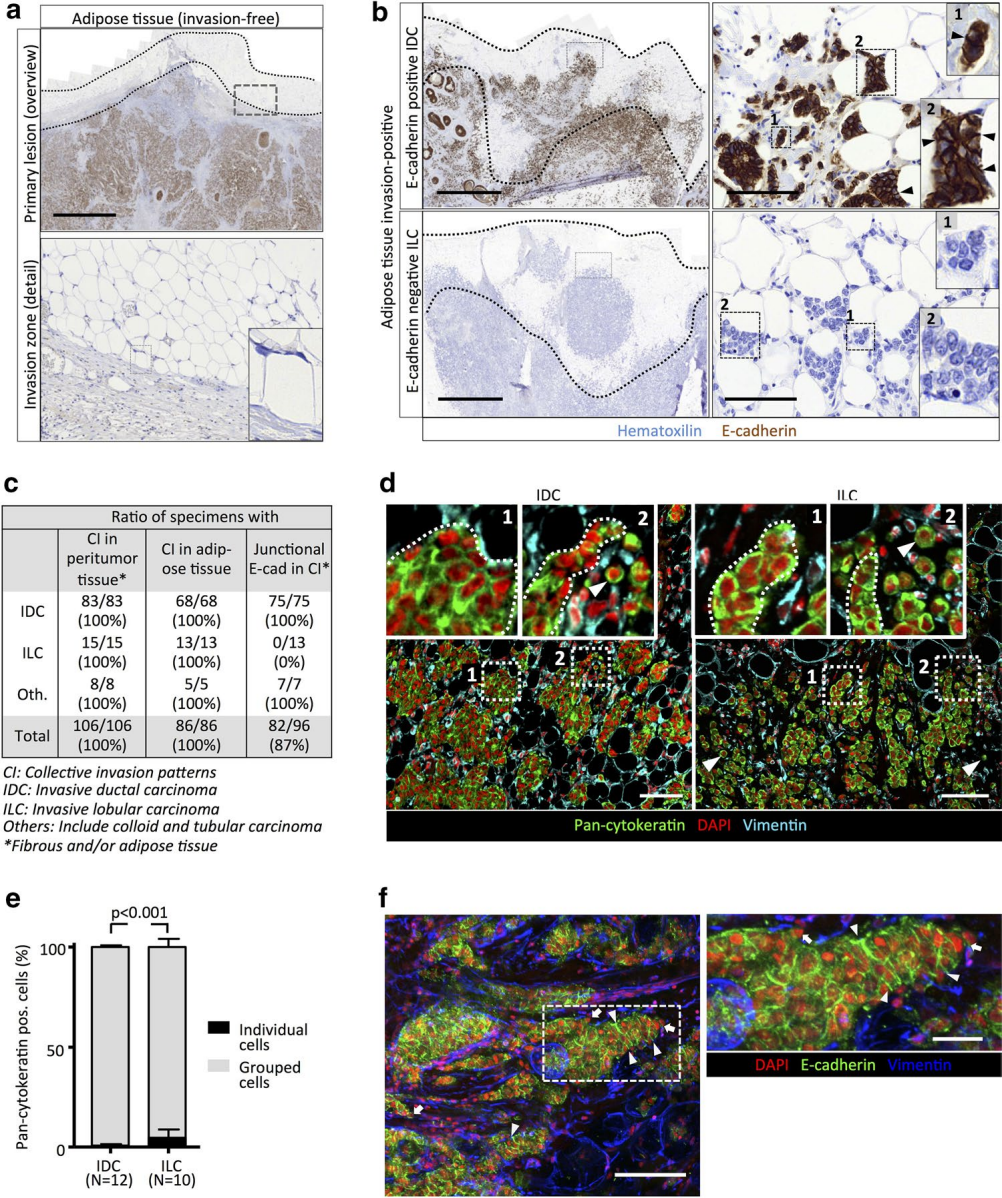
E-cadherin expression and collective invasion patterns in primary breast cancers. **a** Tumor-adipose tissue interface of IDC sample without invasion into the marginal adipose tissue (*black dotted lines*). **b** IDC and ILC invading marginal adipose tissue with and without E-cadherin expression, respectively. *Zooms* show the invasive multicellular cancer cell nests between adipocytes, with *inserts* depicting small (*1*) and large (*2*) cell groups. *Arrowheads* junctional E-cadherin (*brown color*). **c** Absolute numbers and percentage (%) of IDCs and ILCs with peritumor invasion, frequency of multicellular organization of cancer cells within fibrous and/or adipose tissue and frequency of junctional E-cadherin expression. **d, e** Differential distribution of pan-cytokeratin and vimentin in invasive margins of IDC and ILC to discriminate grouped from individualized cell patterns. **d** Representative fluorescent images from IDC and ILC samples (see cohort details and complete data in Supplementary Table 2). *Arrowheads* individual cells; *dashed contour* grouped cells. **e** Quantification of the ratio of collective or individualized pattern of pan-cytokeratin positive cells. Values represent mean percentage of individualized cells ± SD (IDC: 0.75 ± 0.6; ILC: 4.65 ± 4.2). P values, Mann–Whitney test. **f** 3D reconstruction of confocal z-projection (100 μm thickness) from a 200 μm thick IDC sample. *Arrowheads* junctional E-cadherin. *White arrows* leader cells. *Scale bars* 2000 μm (**a, b** overviews); 100 μm (**a, b** details; **d** overview). (Color figure online)

The prevalence of collective invasion in histological primary breast cancer was classified from the presence or absence of multicellular tumor nests (*syn*. clusters) within the fibrous and/or marginal adipose tissue (Fig. 1a). The majority of samples were scored positively for invasion into the peritumor tissue (106/111 samples). All these lesions (106/106), including IDC and ILC, were enriched for multicellular patterns which were detected as (i) multicellular compact strands, (ii) nests or (iii) elongated multicellular chains (Indian files) (Fig. 1b, c; Supplementary Fig. 1a). Using cytokeratin as marker for cells of epithelial origin in the invasion zone, the abundance of collective invasion was confirmed for both E-cadherin positive (IDC) and negative (ILC) samples (Fig. 1d, e; Supplementary Table 2).

### Collective cell invasion in E-cadherin-positive IDC

To verify epithelial collective invasion irrespective of E-cadherin expression, we scored the presence of E-cadherin or, when E-cadherin was not detected, CD44 along cell–cell interactions within multicellular nests and strands in the peri-tumor fibrous or adipose tissue.

In all invasive IDCs (75/75), intercellular E-cadherin with a linear junctional distribution was detected between the majority of cancer cells in the invasion zone, identified by typical histomorphology (Fig. 1a–c), including in very small clusters comprising of 2–6 cells in scattered stromal location (Fig. 1b; Supplementary Fig. 1b). In a subset of samples, E-cadherin intensity was heterogeneous within different positions of the same lesion, with levels ranging from high to moderate (Supplementary Fig. 1b). A subset of 5/75 of IDC samples showed stronger heterogeneity, with up to 1% of tumor cells expressing very low or no detectable junctional E-cadherin (Supplementary Fig. 1b), and 1/75 IDC samples displayed ~5% of the cells that lacked E-cadherin signal at cell–cell contacts (Supplementary Fig. 1c). Thus, IDC retain E-cadherin expression with locoregional expression variability in a minority of lesions, similar to the heterogeneity observed in primary epithelial ovarian cancer tissues [14].

3D reconstruction confirmed multicellular cohesive strands retaining E-cadherin along cell–cell junctions (Fig. 1f; Supplementary information, Movie 1), in line with collective invasion with intact AJs observed in experimental models [11]. When analyzed in a random subset of IDC, a very low frequency (<1%) of individually positioned cytokeratin-positive tumor cells was detected in the invasive regions (Figs. 1d, arrowhead, e; Supplementary Table 2). As a cautionary note, solitary cells identified in thin 2D sections, particularly when located in vicinity of multicellular nests, may still retain contact with multicellular groups located in adjacent section planes, and require detection by 3D reconstruction of serial sections [10].

### Collective cell invasion devoid of adherens junctions in ILC

**Fig. 2:**
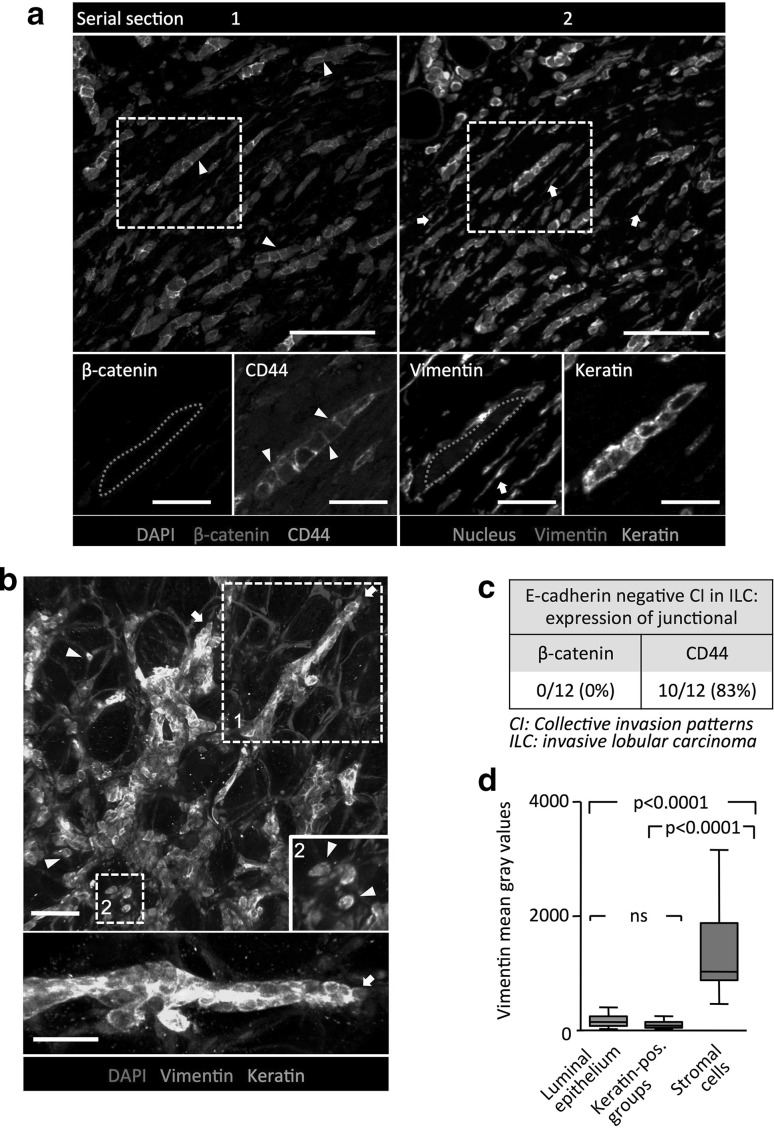
Cell–cell interaction pattern and molecular status of cell–cell junctions in E-cadherin negative invasion zones. **a** Confocal microscopy of β-catenin, CD44, vimentin and epithelial keratins in E-cadherin-negative ILC from two adjacent sections. *White arrowheads* CD44 but not β-catenin localized along cell–cell junctions between keratin positive Indian files. *White arrows* show vimentin-positive stromal fibroblasts. **b** 3D reconstruction of confocal z-projection (80 μm thickness) from a 200 μm thick ILC sample. *White arrowheads* and *arrows depict* individual cancer cells and leader cells, respectively. **c** Molecular characteristics of cell–cell junctions in E-cadherin negative ILC subset, using β-catenin and CD44 as markers. **d** Vimentin levels after densitometric identification of tissue subregions in E-cadherin-negative ILC samples (N = 8). Values represent median (*black line*), 25/75 percentiles (*boxes*) and maximum/minimum values (*whiskers*). P values, one-way ANOVA. *Scale bars* 100 μm (**a, b** overview); 50 μm (**b** detail); 25 μm (**a** detail)

Similar to IDC, the invasive patterns in ILC maintained multicellular organization as strands, clusters and Indian files (Fig. 1b–e; Supplementary Fig. 1a). These multicellular groups lacked intercellular E-cadherin (Fig. 1b, c) and the intracellular AJ adapter protein β-catenin (Fig. 2a, c; Supplementary Fig. 2a, c), confirming the absence of AJs in ILC [15]. Using pan-cytokeratin to identify cells of epithelial origin in ILC samples, 85 to >99% of the tumor cells were part of multicellular groups, with a small fraction of individualized cells (Fig. 1d, e; Supplementary Table 2). Thus, ILC invade predominantly as multicellular files which, compared to IDC, are less cohesive and allow single cell detachment at higher frequency.

CD44 is a cell surface glycoprotein that is ubiquitously expressed in ILC samples [16]. In addition to its canonical role in mediating cell-ECM adhesion, CD44 has been associated with intercellular junctions [17] and implicated in hyaluronan-mediated cell–cell interactions such as between keratinocytes [18] and between endothelial cells and T-cells [19]. We here used CD44 as a marker with stable cell surface localization to denote cell–cell interactions in E-cadherin negative ILC samples. Invasive cells in ILC retained cell–cell juxtaposition with shared linear CD44 staining along cell–cell junctions in 10/12 ILC samples (Fig. 2a, c; Supplementary Fig. 2a, c), whereas 2/12 of the ILC samples lacked CD44 (Supplementary Fig. 2b). The CD44-positive label between cancer cells was present in multicellular nests (Fig. 2a), multilayered strands (Supplementary Fig. 2c) and Indian files (Fig. 2a; Supplementary Fig. 2a). In contrast to IDC, which formed thicker strands, AJ-negative multicellular files in ILC were thinner, with a thickness of 1–3 adjacent cells (Fig. 2b; Supplementary information, Movie 2). Thus, multicellular cancer groups in ILCs lack adherens junctions but retain CD44 between juxtaposed neighbor cells. This indicates that ILCs invade the peritumor tissue predominantly as collective files.

### Lack of vimentin in collective invasion fronts

We next addressed whether collective invasion patterns demonstrate features of EMT, including the downregulation of epithelial keratins and upregulation of vimentin [4]. Both E-cadherin positive and E-cadherin negative collective invasion patterns in IDC and ILC, respectively, expressed pan-cytokeratin, with intensities similar to the normal luminal epithelium (Fig. 2a, b; Supplementary Fig. 2), and lacked vimentin expression (Fig. 2a, d; Supplementary Fig. 2b-e). Vimentin was present in the spindle-shaped cells in the peritumor stroma (Fig. 2a; Supplementary Fig. 2c), which are commonly recognized as stromal fibroblasts [20] or further may represent cancer cells that have undergone complete EMT [21]. These results indicate that multicellular groups in both IDC and ILC retain epithelial characteristics and lack traits of complete EMT.

### Association of collective cancer invasion and distant metastasis

To address the extent of collective invasion two complementary strategies were used. The areas of the marginal adipose tissue occupied by collective invasion fronts were histomorphologically scored by a breast cancer pathologist and compared for patient subsets with (n = 44) or without (n = 58) distant metastasis within 5 years of follow-up.

**Fig. 3:**
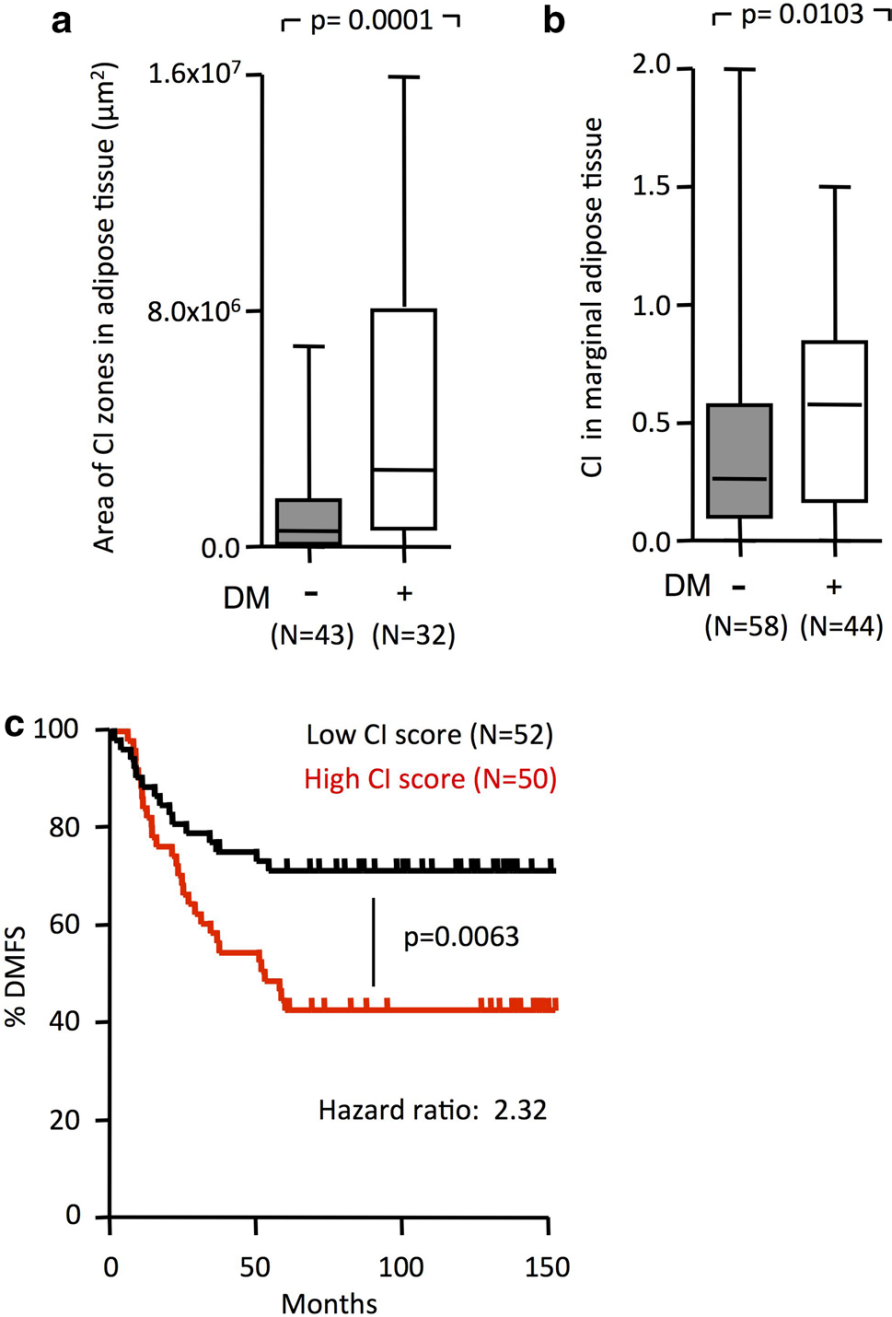
Correlation of collective cancer invasion into the adipose tissue with distant metastasis. Collective invasion score in adipose tissue, calculated by **a** image analysis of IDC samples, using E-cadherin for surface mapping, and **b** visual pathological inspection in patient subgroups without and with distant metastasis (DM) from the cohort including IDC and ILC (102 patients that contained adipose tissue in the tumor section) within 5 years of follow-up. Values in **a, b** display medians (*black line*), 25/75 percentiles (*boxes*) and maximum/minimum values (*whiskers*). P values, Mann–Whitney test. **c** Kaplan–Meier survival plot comparing distant metastasis free survival (DMFS) between patients with high versus low histopathological collective invasion scores in the adipose tissue using the analysis groups shown in (**b**). P value and hazard ratio with 95% confidence interval, Log-rank test

In addition, for IDC samples, the histomorphological score for collective invasion was validated by semi-automated quantification of collective invasion, using E-cadherin as reference marker for the invasion zone. The CI scores obtained by both strategies showed strong positive correlation (Supplementary Fig. 3b), indicating comparable precision for quantifying epithelial collective invasion. Both scoring approaches revealed that the extent of collective invasion into the adipose tissue correlated positively with distant metastasis (Fig. 3a, b). Consistently, distant metastasis-free survival was significantly reduced in patients with high CI scores (Fig. 3c; Supplementary Fig. 3c) with respective hazard ratios of 2.32 and 2.29 using histomorphological scoring and segmentation-based analysis. The CI score neither correlated with lymph node metastasis, tumor grade, tumor size or menopausal status (Supplementary Fig. 3d). Thus, the extent of collective invasion in the marginal adipose tissue is associated with metastasis outcome in breast cancer patients.

## Discussion

Using cell–cell adhesion markers as basic criteria for collective migration [8], we here identified collective invasion as the predominating strategy for local tissue infiltration in primary breast cancer, irrespective of the histological subtype and E-cadherin status. The association of collective adipose tissue invasion with metastatic disease suggests an important role for cell–cell cooperation in systemic dissemination.

### Collective invasion with and without adherens junctions

In moving epithelia, stable cell–cell junctions maintained by E-cadherin are critical in mediating multicellular coordination and polarity, mechanotransduction and movement [6, 22]. Here, we could discriminate at least two types of collective invasion using cytokeratin as an epithelial identifier together with AJ markers and vimentin as EMT and stromal marker: (i) E-cadherin positive and vimentin-negative compact strands in IDC and (ii) thinner and arguably more loose files in ILC, which retain cell–cell interactions but lack both E-cadherin and vimentin. In IDC, all lesions and, per lesion, the vast majority of cells retain E-cadherin expression, consistent with collective epithelial invasion [8]. The invasive patterns in ILC, although predominantly collective, show higher probability of cell individualization than in IDC. This suggests that both collective and single-cell invasion occur in parallel in ILC and may provide a broader range of metastatic phenotypes. In ILC, CD44 was used to visualize regions of overlapping membrane staining in juxtaposed cells. The pattern of CD44-positive cell–cell interactions in ILC is consistent with an experimental ILC mouse model after somatic inactivation of the E-cadherin gene, where invading cells retain multicellular organization instead of disseminating individually [23]. Whether CD44 mediates cell–cell adhesion during collective invasion of ILC or rather acts as functionally inert marker for cell–cell interactions remains to be clarified. Both CD44 and hyaluronic acid are expressed in breast cancer cells, thus CD44 could potentially engage to hyaluronic acid present at the counterpart membrane of the contacted cell [24]. In T cells, CD44 supports cell–cell adhesion, via hyaluronic acid, to the endothelium and initiates transendothelial migration [19, 25], and cell–cell interactions may additionally be supported via engagement of a heparan and chondroitin sulfate proteoglycan homologue of CD44 between adjacent cells [18]. Beyond CD44, other adhesion systems, including immunoglobulin family members (e.g. LCAM, ALCAM, L1-CAM, NCAM) may support cell–cell interactions in the absence of E-cadherin in ILC [26]. Cell–cell junctions in ILC are likely sufficiently stable to mediate cell–cell binding when tissue density is high and extracellular confinements force cells together (“cell-jamming”) [27].

The molecular variability of collective patterns suggests that local tissue penetration represents a continuum from quiescent epithelium to multicellular epithelial invasion [28], recapitulating variants of collective invasion during tissue morphogenesis and regeneration [5]. Likely, such variety of collective invasion patterns is relevant for other epithelial neoplasms, including colorectal and pancreatic cancer [10].

### Collective invasion as initiator of cancer metastasis

Invasion of breast cancer cells into the adipose tissue correlates with disease progression and poor clinical outcome, including lymph node metastasis and disease-free survival [1]. The here identified association between the extent of collective cancer invasion into the adipose tissue and the appearance of distant metastasis supports concepts on tumor-cell cooperation [6] and paracrine adipose tissue functions in enhancing metastasis [29]. In this cohort study CI scores did not correlate with other known prognostic factors, including tumor grade and size. This may indicate the CI score as an independent prognostic parameter, which requires verification by an independent dataset. The CI score, which here was obtained using clinical routine samples, was not affected by variations of the region of adipose tissue contained in each sample. The CI score will be amenable to routine histopathological analysis, alongside with currently used prognostic parameters [30] to identify particularly high-risk patient subsets.

Collective invasion into the peritumor stroma may support distant metastasis by several mechanisms, including pro-survival, pro-invasive and mitogenic signals provided by cell–cell interactions and paracrine growth factors released between tumor cells [31]; its contribution to desmoplasia-like ECM remodeling [32]; and by enabling a multicellular mass with high mechanical stability during both tissue penetration and hematogenous spread [7]. In addition, tumor-associated adipocytes express migration-enhancing ECM components and soluble factors, which may promote tumor cell migration and intravasation by supporting angiogenesis and increasing vessel permeability [29]. Tumor-cell and adipocyte-derived paracrine signaling may thus cooperate to enhance collective metastasis.

## Implications

The range of collective invasion patterns detected in IDC and ILC indicates remarkable morphologic and molecular diversity of collective behaviors. Defining E-cadherin-dependent and -independent types of cell–cell cooperation and their cross-talk with EMT and other activation programs will be required to define the subtypes, mechanisms and interconversions of collective cooperation in epithelial malignancies.

## Electronic supplementary material

Below is the link to the electronic supplementary material.

Supplementary material 1 (DOCX 35 KB)

Supplementary material 2 (JPG 2539 KB)

Supplementary material 3 (JPG 1746 KB)

Supplementary material 4 (JPG 868 KB)

Supplementary material 5 (AVI 28114 KB)

Supplementary material 6 (AVI 22947 KB)

## Abbreviations

IDC: Invasive ductal carcinoma
ILC: Invasive lobular carcinoma
EMT: Epithelial-to-mesenchymal transition
AJ: Adherens junction
