# Supplementary material for: Collective invasion in ductal and lobular breast cancer associates with distant metastasis
Source: Clin Exp Metastasis. 2017 Sep 11;34(6):421–9. doi: 10.1007/s10585-017-9858-6 (PMC5711975; doi:10.1007/s10585-017-9858-6)
Supplement: Supplementary file 1 — Supplementary material 1 (DOCX 35 KB) [file 10585_2017_9858_MOESM1_ESM.docx]

**Supplementary information for:**

**Collective invasion in ductal and lobular breast cancer samples associates with distant metastasis**

Antoine A. Khalil^1^, Olga Ilina^2^, Pavlo G. Gritsenko^2^, Peter Bult^3^,

Paul N. Span^4^, Peter Friedl^1,2,5,6^

^1^ Department of Dermatology and Graduate School of Life Science, University of Würzburg, Würzburg, Germany

^2^ Department of Cell Biology, Radboud University Medical Center, Nijmegen, The Netherlands

^3^ Department of Pathology, Radboud University Medical Center, Nijmegen, The Netherlands

^4^ Department of Radiation Oncology, Radboud University Medical Center, Nijmegen, The Netherlands

^5^ David H. Koch Center for Applied Genitourinary Cancers, The University of Texas MD Anderson Cancer Center, Houston 77070, Texas, USA

^6^ Cancer Genomic Centre, 3584 CG Utrecht, The Netherlands

Correspondence: peter.friedl@radboudumc.nl

**Supplementary Figures**

**Supplementary Figure 1. Morphological mapping of IDC and ILC invasion and E-cadherin heterogeneity.** (a) Multicellular organization in IDC and ILC. Representative histology of IDC and ILC (H&E staining). Dashed lines highlight morphological variations of multicellular tumor cell groups. (b, c) Variability of E-cadherin expression levels in collective invasion zones in IDC samples with rare E-cadherin-low to -negative events. Examples for variable E-cadherin expression ranging from strongly positive staining to locally diminished levels along cell-cell junctions, with less than 1% (b, representing 5/75 samples) or 5% of cancer cells with E-cadherin-low to -negative cell-cell junctions (c, representing 1/75 samples). Zooms, invasive subregions with varying junctional E-cadherin levels, with red asterisks denoting negative status, white arrowheads denoting low and black arrowheads denoting moderate/high E-cadherin intensity. Scale bars, 2000 μm (a-c, overview), 100 μm (a, detail), 50 μm (b, c, detail).

**Supplementary Figure 2. Molecular mapping of ILC invasion.**

(a) Organization of cell-cell junctions in ILC. Confocal microscopy of β-catenin, CD44 and epithelial keratins. White arrowheads denote the presence of both β-catenin and CD44 localized along cell-cell junctions in the luminal epithelium (blue dashed box) and absence of β-catenin with retained CD44 in indian files (green dashed box). (b) Confocal microscopy of CD44-negative but keratin-expressing cancer cells in ILC, representing 2/12 ILC samples. Dashed lines, cell clusters; arrowheads, individualized cells. (c) Lack of vimentin in adherens junctions-negative ILC clusters. Arrowheads, CD44 localized along cell-cell junctions; Arrow, vimentin-positive stromal cells. (d) Spatial and molecular segregation of epithelial and stromal cells. Densitometric analysis of vimentin and pankeratin intensity in ILC samples. Color scheme as in (e), with each symbol representing each parameter per sample. Variations in parameter number reflect 5 samples which only contained tumor and lacked luminal epithelium of normal ducts. (e) Vimentin levels in tumor and stromal compartments of IDC samples defined by cytokeratin status. Sample numbers: N=8 (luminal epithelium) or 13 (groups, stromal cells). P values, one-way Anova. Scale bars, 100 μm (a, b, c), 25 μm (a, c, detail).

**Supplementary Figure 3.** **Validation of collective invasion scoring strategies and association of CI score with clinical parameters.** (a) Adipose tissue area fraction per sample obtained by visual scoring in patient subgroups with and without distant metastasis. (b) Linear regression analysis comparing the collective invasion (CI) scores obtained by visual pathological scoring or semi-automated image analysis in E-cadherin positive IDC sample subset (n=75). P values, Spearman correlation. (c) Kaplan-Meier survival plot predicting distant metastasis free survival (DMFS) for patients with high versus low areas of collective invasion zones obtained by digital image analysis. P value and hazard ratio with 95% confidence interval, Log-rank test. (d) Collective invasion score in the adipose tissue obtained by histopathological scoring relative to other stratifiers of patient subsets, including menopausal status, tumor size, tumor grade and lymph node metastasis at time of surgery. Values in (a, d) represent medians (black line), 25/75 percentiles (boxes) and maximum/minimum values (whiskers). P values (a, d), Mann Whitney test.

**Supplementary movies**

**Supplementary information, Movie 1.** 3D reconstruction of invasive ductal carcinoma as E-cadherin positive collective invasion strands (100 µm z-stack, 2.5 µm step size). Stromal cells and adipocytes are visualized using vimentin staining. Scale bars, 100 µm (overview), 50 µm (detail). Related to Fig. 1.

**Supplementary information, Movie 2.** 3D reconstruction of invasive lobular carcinoma, using cytokeratin to identify epithelial origin of collective strands (80 µm z-stack, 2.5 µm step size). Stromal fibroblasts and adipocytes are visualized using vimentin staining. Scale bars, 100 µm (overview), 50 µm (detail). Related to Fig. 2.

**Supplementary Table 1: Patient stratification based on lymph node metastasis**

|  | DM-Free | DM |
| --- | --- | --- |
| Frequency of LNM | 31/63 (49%) | 26/48 (54%) |

*LNM: Lymph node metastasis; DM: Distant metastasis.*

*Percent values indicate the LNM-positive fraction in each patient subset.*

| Sample | IDC/ILC | Pan cytokeratin positive cells | | | |
| --- | --- | --- | --- | --- | --- |
|  |  | Total number of cells | Number of individual cells | % Individual cells | % Grouped cells |
| 1 | IDC | 574 | 1 | 0.2 | 99.8 |
| 2 | IDC | 187 | 4 | 2.1 | 97.9 |
| 3 | IDC | 411 | 2 | 0.5 | 99.5 |
| 4 | IDC | 734 | 5 | 0.7 | 99.3 |
| 5 | IDC | 571 | 5 | 0.9 | 99.1 |
| 6 | IDC | 431 | 2 | 0.5 | 99.5 |
| 7 | IDC | 589 | 2 | 0.3 | 99.7 |
| 8 | IDC | 542 | 4 | 0.7 | 99.3 |
| 9 | IDC | 332 | 1 | 0.3 | 99.7 |
| 10 | IDC | 343 | 6 | 1.7 | 98.3 |
| 11 | IDC | 397 | 2 | 0.5 | 99.5 |
| 12 | IDC | 611 | 0 | 0.0 | 100.0 |
| 13 | ILC | 341 | 14 | 4.1 | 95.9 |
| 14 | ILC | 194 | 13 | 6.7 | 93.3 |
| 15 | ILC | 270 | 10 | 3.7 | 96.3 |
| 16 | ILC | 339 | 22 | 6.5 | 93.5 |
| 17 | ILC | 232 | 35 | 15.1 | 84.9 |
| 18 | ILC | 374 | 14 | 3.7 | 96.3 |
| 19 | ILC | 292 | 10 | 3.4 | 96.6 |
| 20 | ILC | 594 | 2 | 0.3 | 99.7 |
| 21 | ILC | 537 | 5 | 0.9 | 99.1 |
| 22 | ILC | 405 | 8 | 2.0 | 98.0 |

**Supplementary Table 2: Absolute numbers and percentages of individual and grouped pan-cytokeratin positive cells**

*IDC: Invasive ductal carcinoma; ILC: Invasive lobular carcinoma*
