# Supplementary figures and images for: Collective invasion in ductal and lobular breast cancer associates with distant metastasis
Source: Clin Exp Metastasis. 2017 Sep 11;34(6):421–9. doi: 10.1007/s10585-017-9858-6 (PMC5711975; doi:10.1007/s10585-017-9858-6)

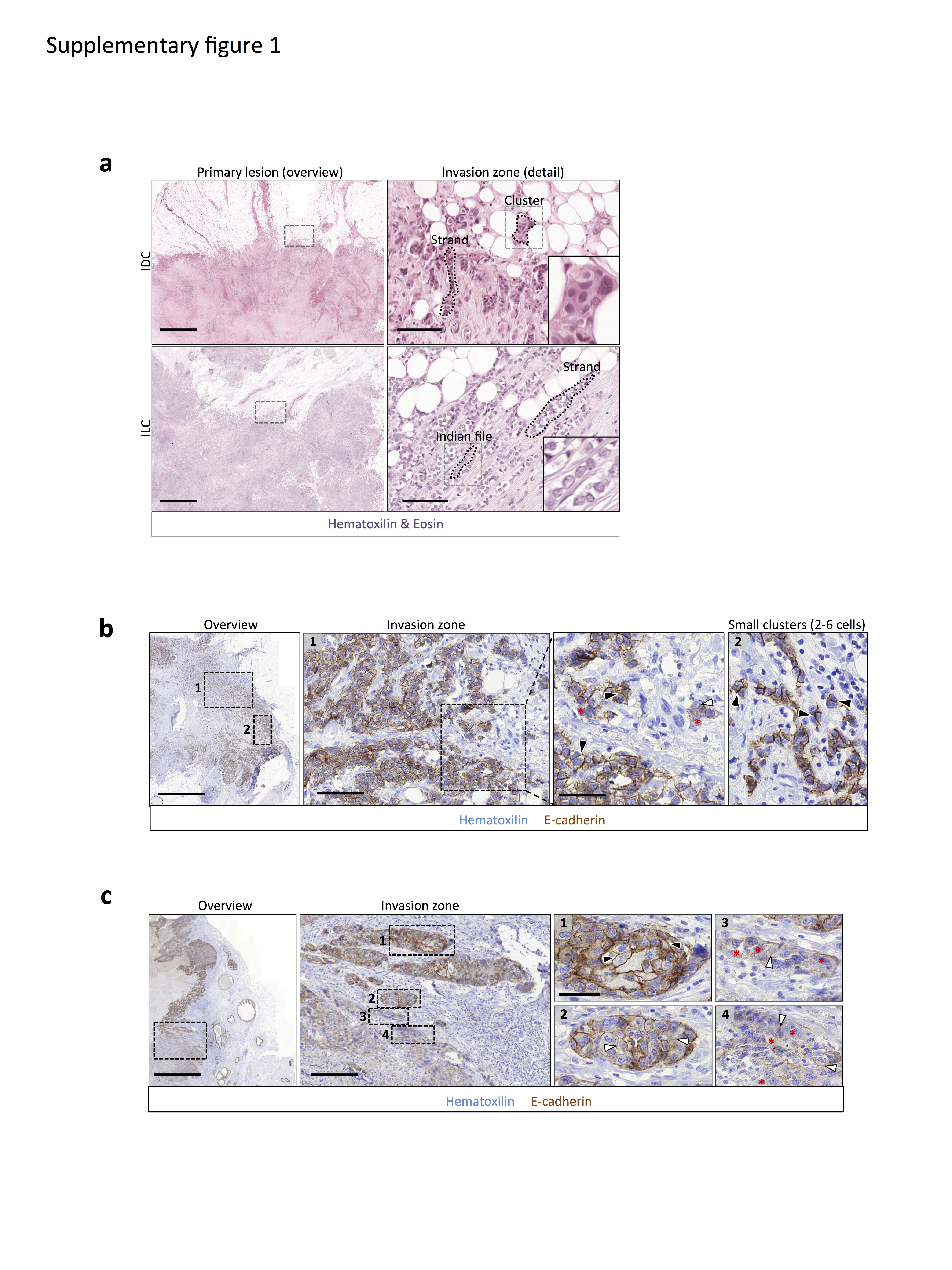

Supplement: Supplementary file 2 — Supplementary material 2 (JPG 2539 KB) [file 10585_2017_9858_MOESM2_ESM.jpg]

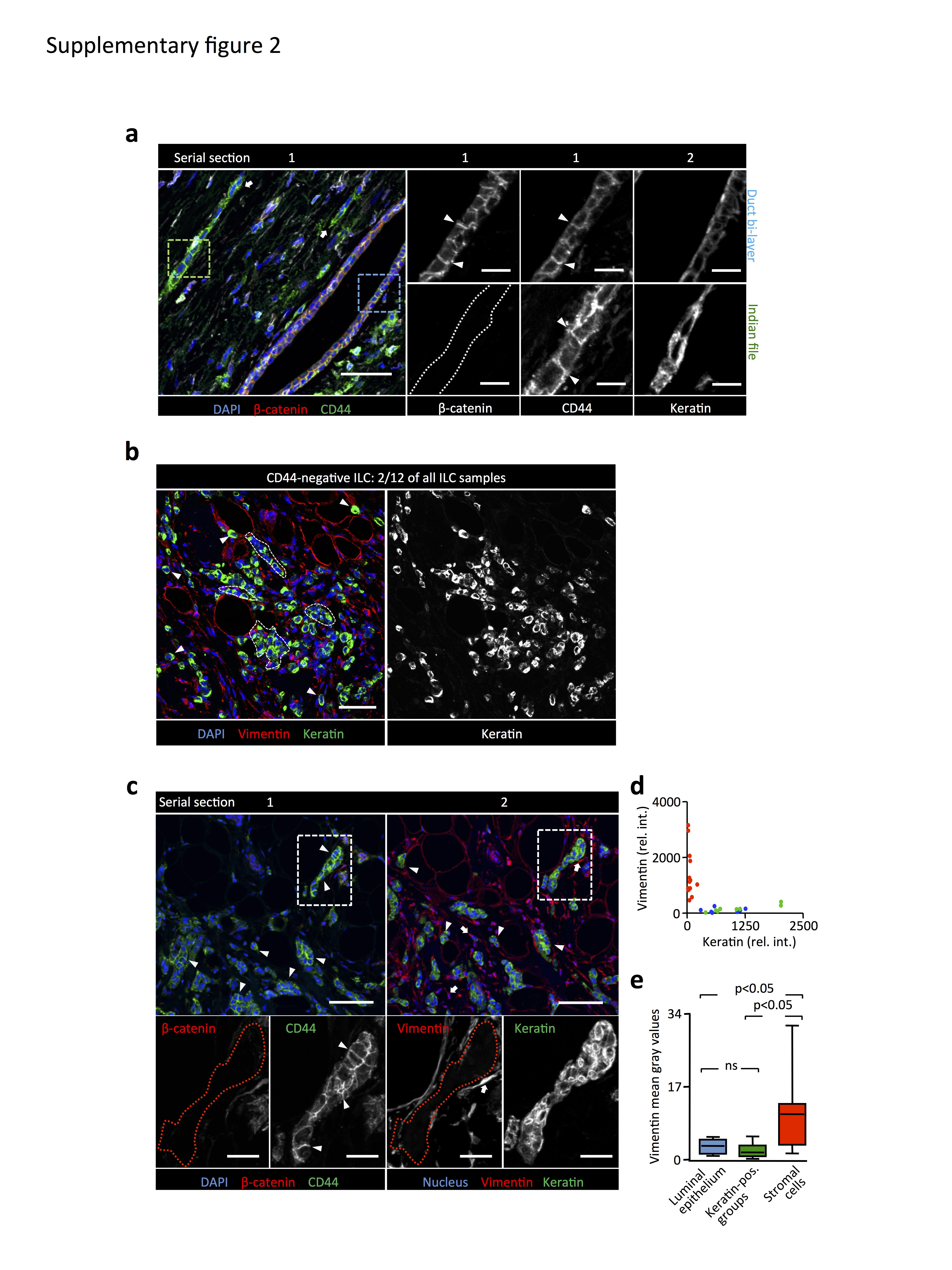

Supplement: Supplementary file 3 — Supplementary material 3 (JPG 1746 KB) [file 10585_2017_9858_MOESM3_ESM.jpg]

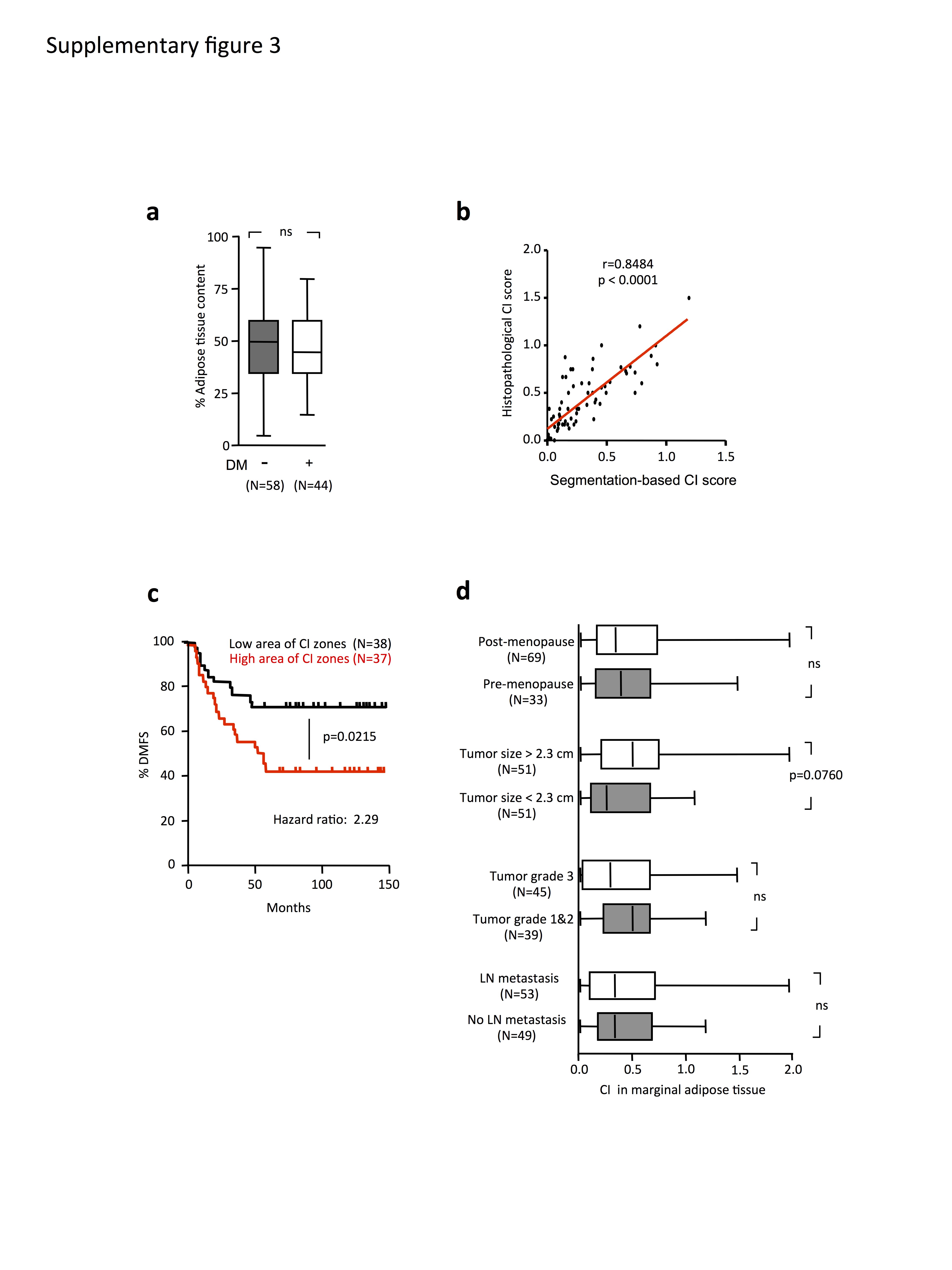

Supplement: Supplementary file 4 — Supplementary material 4 (JPG 868 KB) [file 10585_2017_9858_MOESM4_ESM.jpg]
